# Perception of COVID-19 and Vaccine Acceptance among Healthcare Workers

**DOI:** 10.1155/2022/1607441

**Published:** 2022-12-02

**Authors:** Nader Nemr, Rania M. Kishk, Nourhan Hassan Soliman, Rasha Mohammed Farghaly, Safaa M. Kishk, Nageh Louis

**Affiliations:** ^1^Endemic and Infectious Diseases Department, Faculty of Medicine, Suez Canal University, Ismailia 41522, Egypt; ^2^Microbiology and Immunology Department, Faculty of Medicine, Suez Canal University, Ismailia 41522, Egypt; ^3^Clinical Pathology Department, Faculty of Medicine, Suez Canal University, Ismailia 41522, Egypt; ^4^Community, Occupational and Environmental Medicine Department, Faculty of Medicine, Suez Canal University, Ismailia 41522, Egypt; ^5^Pharmaceutical Medicinal Chemistry Department, Faculty of Pharmacy, Suez Canal University, Ismailia 41522, Egypt

## Abstract

**Background:**

COVID-19 infection is more likely to be acquired and transmitted by healthcare workers (HCWs). Furthermore, they serve as role models for communities in terms of COVID-19 vaccination attitudes. As a result, HCWs' reluctance to vaccinate could have a significant impact on pandemic containment efforts.

**Aim:**

To characterize the current COVID-19 vaccine approval situation among healthcare workers and to determine the most likely reason for agreement or disagreement with COVID-19 vaccination.

**Methods:**

This cross-sectional design included 451 HCWs from COVID-19 treatment institutions, with COVID-19 exposure risk changing depending on job function and working location.

**Results:**

The study recruited 156 physicians and 295 nurses, of whom 58.1% were female and 41.9% were male. Physicians had a significantly higher rate of participation in COVID-19 pandemic prevention and control, with a rate of 69.9% versus 55.3% of nurses. Acceptance of COVID-19 vaccination was reported by 40.8% of HCWs. The rate of acceptance was significantly higher among physicians (55.1%) than among nurses (33.2%) (*p* < 0.001). Most HCWs (67.8%) believed the vaccine was not effective. Physicians showed more significant trust in the effectiveness of the vaccine than nurses (41% and 27.5, respectively) (*p*=0.003). Concerning vaccine safety, only 32.8% of HCWs believed it was safe. This was significantly higher in physicians (41.7%) than in nurses (28.1%) (*p*=0.004).

**Conclusion:**

Vaccination uncertainty is common among healthcare personnel in Egypt, and this could be a significant barrier to vaccine uptake among the public. Campaigns to raise vaccine knowledge are critically needed.

## 1. Introduction

Coronavirus disease 2019, also known as COVID-19, is a rapidly spreading pandemic caused by a novel human coronavirus called SARS-CoV-2, an enveloped single-stranded RNA virus that was previously known as 2019-nCov [1–3]. It was first reported in December 2019 among patients with viral pneumonia in Wuhan, China, as the world's most serious health issue [4].

On January 30, 2020, World Health Organization (WHO) proclaimed this extremely contagious virus a “public health emergency of international concern” due to its rapid spread across many countries. COVID-19 was later declared a global pandemic by the WHO on March 11, 2020, due to an increase in the number of affected countries, cases, and deaths [5].

Health Care Workers (HCWs) are on the front lines of COVID-19 pandemic defense, and they are vulnerable to not only COVID-19 infection but also psychological distress, long working hours, fatigue, occupational stigma, and physical violence [6, 7]. The WHO recommends that HCWsand patients' close contacts be protected to prevent the disease from spreading. Overcrowding, a lack of isolation facilities, and a contaminated environment all contribute to disease transmission among HCWs, which is likely aided by HCWs' lack of understanding and awareness of infection control procedures [8]. Inadequate expertise and attitudes among HCWs can have a direct impact on practices, resulting in delayed diagnosis and poor infection control practices [9, 10]. Hand washing, social distancing, and respiratory hygiene (covering mouth and nose while coughing or sneezing) are all primary preventive measures [11]. It would be beneficial to protect these healthcare workers from COVID-19 infectious illness, not just for themselves but also for their family members as well as their patients.

As the COVID-19 outbreak poses a serious threat to public health [12], researchers were racing to develop and test COVID-19 vaccines [13]. A COVID-19 vaccine has been considered necessary to end the pandemic, and multiple experimental trials to develop a COVID-19 vaccine are currently being coordinated at a higher level [12]. Following vaccine development, COVID-19 vaccination programs faced the challenge of gaining community acceptance.

Vaccine development took years in the past. Despite its availability, public acceptability of a novel COVID-19 vaccine created in a short period of time remains dubious [14]. Lessons gathered from earlier influenza pandemics, when the vaccine was first introduced and acceptance rates were variable in many countries, necessitate a thorough knowledge of the vaccination reluctance problem [15, 16].

The launch of the COVID-19 vaccine rollout in December 2020 marked a turning point in the fight against this pandemic [17]. Several COVID-19 vaccines are widely used throughout the world, including mRNA vaccines (Pfizer BioNTech, Moderna, and Johnson & Johnson), viral vector vaccines (AstraZeneca and Sputnik V), and inactivated vaccines (Sinopharm, Sinovac, and COVAXIN) [18].

The WHO approved the Pfizer COVID-19 vaccine (BNT162b2) for emergency use on December 31, 2020. On February 15, 2021, the Serum Institute of India and SKBio produced the AstraZeneca/Oxford COVID-19 vaccine. On March 12, 2021, Janssen (Johnson & Johnson) and Moderna released the Ad26.COV2.S vaccine [19].

The Egyptian Ministry of Health (MOH) gave healthcare personnel top priority when the COVID-19 vaccine became accessible in Egypt [20]. They posed the greatest risk of contracting the novel virus of any group. The first wave of immunized HCWs received the Oxford-AstraZeneca COVID-19 vaccine until all doses were used up, at which point they switched to the Sinopharm and Sinovac vaccines [21, 22]. The WHO estimates that Egypt has 513,790 confirmed COVID-19 cases up through May 2022, including 24,641 fatalities, and that 82,017,392 vaccine doses were administered in total [23]. Coronavirus vaccinations were found to be well tolerated and safe, with the majority of postvaccination adverse effects being mild to moderate, according to a research study of COVID-19 vaccine side effects among the Egyptian population [24].

The reasons for vaccine rejection vary, but vaccination hesitancy is a widespread occurrence worldwide [11–13]. Perceived hazards vs. advantages and a lack of knowledge and awareness were among the most prevalent causes [25, 26]. When it comes to COVID-19 vaccination reluctance, there is a substantial correlation between wanting to receive the vaccine and its perceived safety [27], as well as between having a bad opinion of the vaccine and a desire not to receive it [28].

Due to the complexity of this phenomenon, research on the worldwide effects of vaccination hesitancy, especially readiness to adopt COVID-19 vaccines, may be limited. This implies that vaccine reluctance is influenced by cognitive, psychologic, sociodemographic, and cultural factors [29, 30].

The Advisory Committee on Immunization Practices (ACIP) recommended that HCWs receive immunization priority in December 2020 [31, 32]. A study was conducted to describe the potential COVID-19 vaccine acceptance among Egyptian healthcare providers, in which 45.9% accepted to receive the vaccine, while 40.9 percent refused to take the vaccine [33]. They concluded that Egyptian HCWs willingness to accept COVID-19 vaccination is lower than in western nations but higher than in African countries. Egyptian HCWs' vaccine apprehension could be a major factor in the COVID-19 approval decision [34]. The fact that HCWs are exposed to more professional information and subsequently have more concerns about the efficacy and safety of the COVID-19 vaccine may be a contributing factor to this finding regarding the intention of COVID-19 vaccination among healthcare workers. This concern may affect HCWs' decisions to get vaccinated and prevent them from recommending vaccination to patients [32].

In 2019, the World Health Organization (WHO) listed ten global health hazards, including vaccine indecision and the possibility of a pandemic [35]. Both threats actually pose a threat to the world. The term “vaccine hesitancy” refers to a delay in receiving a vaccine, vaccine acceptance or denial, regardless of accessibility of vaccination programs [36]. Indecisiveness over vaccines is also worrying for public health doctors [37, 38] and nurses [39].

Our study aimed to characterize the current COVID-19 vaccine acceptance among HCWs and determine the most likely reasons for agreement or disagreement with COVID-19 vaccination. Also, we aimed at determining healthcare workers' perceptions and attitudes, identifying the elements that influence their attitudes, and identifying factors that could help enhance vaccine acceptance among healthcare professionals. This report serves as guidance for Egyptian health officials and public health specialists, highlighting the COVID-19 vaccine's expected problems among HCWs.

### 1.1. Study Objectives

#### 1.1.1. Primary Objectives

- Measuring the rate of acceptance of COVID-19 vaccination among healthcare workers.

#### 1.1.2. Secondary Objectives

- Describe causes of refusal of COVID-19 vaccination through answering a questionnaire of the causes of avoidance of vaccine
- Identify the needed strategies to improve vaccine acceptance among healthcare workers

## 2. Subjects and Methods

### 2.1. Study Design

This study was a cross-sectional, descriptive, online-based study carried out in Egypt from January 2021 to May 2021. Our study was conducted on a representative sample of HCWs(doctors, nurses, laboratory workers, and technicians) who are working in Ismailia and Suez Governorates, Egypt.

### 2.2. Ethics

Approval was obtained from the Ethics Committee of the Suez Canal University Faculty of Medicine. The procedures utilized in this study adhere to the tenets of the Declaration of Helsinki.Participants' agreement to participate in the study was based on their completion of the questionnaire (Reference: Research#4553).

### 2.3. Data Collection

#### 2.3.1. Sample Size Calculation

Sample size was 451 calculated according to the following equation: *n* = (Z*α*/2/*E*)2 *∗* *p*(1 − *p*), where *n* = sample size; *Z α*/2 = 1.96 (the critical value that divides the central 95% of the *Z* distribution from the 5% in the tail); *p* = the prevalence of COVID-19 vaccine acceptance = 36% [40]; *E* = precision = 5%.

#### 2.3.2. Sampling Approach

A snowball technique was used for data collection.

### 2.4. Data Collection Tool

The questionnaire created on Google Forms was distributed via social media and WhatsApp groups. The questionnaire was completed by 451 healthcare professionals and took about 4 minutes to complete. The survey's inclusion criteria were listed in the consent form at the beginning of the questionnaire. Participation in this study was completely voluntary, and participants were not compensated in any way. During the data collection process, participants' anonymity was ensured.

### 2.5. Data Collection Process

#### 2.5.1. Translation and Piloting

Closed-ended questions in English and Arabic were included in the online poll (for nurses, laboratory workers, and technicians). It was pilot tested on ten people to ensure that the questionnaire was clear, and the results were not included in the final dataset. Potential respondents were sent a link to Google Forms.

For each item on the questionnaire, a forward and backward translation was performed. The scales were translated from English to Arabic by one translator, and back translation was conducted by another. Disparities between the original English and the translated versions were settled through consensus.

#### 2.5.2. Questionnaire and Data Collection

The data were collected using an electronic questionnaire. Participants completed a Google form's questionnaire by April 2021. The sample was representative of all Egyptian healthcare workers in terms of age, gender, and occupation.

The following data were collected including

1. Baseline demographic information, such as occupation and the presence of co-occurring disorders
2. Inquire whether the participants participate in the prevention and control of epidemic, receive other vaccines in the past 3 years, receive seasonal influenza vaccine in the last 3 years or get COVID-19 infection
3. Assessment of self-perceived risk of COVID-19 and acceptance of the COVID-19 vaccine
4. Assessment of behaviors postepidemic (post-COVID-19)
5. Prior to data collection, participants gave their informed consent

### 2.6. Data Management and Statistical Analysis

Completed forms were imported into a Microsoft Excel spreadsheet. SPSS version 23 was used to collect, tabulate, and statistically analyze all data. Absolute frequencies (number) and relative frequencies (percentage) were used to express categorical qualitative variables (percentage). The chi-square test (*χ*2test) was used to compare categorical data. Fisher's exact test was used for tables with ≥20% of cells having an expected value <5. The Mann–Whitney test was used for comparing quantitative nonparametric variables among groups. A *p* value of >0.05 was considered statistically significant (S).

## 3. Results

The study included 451 HCWs (156 physicians and 295 nurses); 58.1% of them were female, and 41.9% were male. Most of the HCWs were from urban residences (79.2%), while only 20.8% were from rural ones. Regarding marital status, 59.2% were married, 35% were single, 4% were divorced, and 1.8% were widows. The number of household members for the studied HCWs ranged from 1 to 10 with a median of 4 persons. Concerning the HCWs' level of education, 30.2% had a bachelor's degree, 27.3% had a diploma, 19.7% had a master's degree, 18.2% had completed a course in a nursing institute, and only 4.7% had a medical doctorate. The HCWs' duration of work ranged between 1 and 42 years with a mean of 8 ± 6.5 (Table 1).

As shown in Table 2, the most frequently reported chronic illnesses were diabetes and hypertension, which were highly reported among nurses than physicians.

Table 3 shows that physicians had a significantly higher rate of participation in COVID-19 pandemic prevention and control with a rate of 69.9% versus 55.3% of nurses. There was no significant difference between physicians and nurses regarding the COVID-19 infection rate 55.1% of physicians and 51.9% of nurses. Concerning the methods of COVID-19 diagnosis among HCWs, clinical presentation was the highest reported method (42.4% of HCWs), and it was more significantly reported by physicians than among nurses (*p* < 0.001). The least reported method was PCR, although it was significantly more reported among physicians than among nurses (*p*=0.020).

Table 4 shows the knowledge, attitude, and practices of HCWs towards COVID-19 disease. There was a significantly better knowledge base among physicians than nurses regarding the COVID-19 infection.

As shown in Table 5, 48.8% of HCWs stated that they are at risk of getting COVID-19 infection, but they believe they will get mild symptoms which will not require hospitalization. Only 5.1% of HCWs think they will not get reinfected after recovering from a confirmed infection with COVID-19. There was a statistically significant difference between nurses' and physicians' responses, where 12.2% of nurses were confident that they will not get infected, while only 6.4% of physicians had the same response. In addition, 6.8% of nurses believed they would not get reinfected after recovering from a confirmed COVID-19 infection, while only 1.9% of physicians recorded this belief. On the other hand, 57.7% of physicians believed they will have mild symptoms if got COVID-19 infection versus 44.1% of nurses.

Most of the physicians (55.1%) accept to take COVID-19 vaccines than nurses (33.2%) (*p* < 0.001); also, vaccination campaign organized by the hospital was the preferred place for getting the vaccine (Table 6).

Table 7 shows HCWs' beliefs towards the COVID-19 vaccine. Most HCWs (67.8%) believed that the vaccine was not effective. Physicians showed more significant trust in the effectiveness of the vaccine than nurses (41% and 27.5%, respectively) (*p*=0.003). Concerning vaccine safety, only 32.8% of HCWs believed it was safe. This was significantly higher in physicians (41.7%) than in nurses (28.1%) (*p*=0.004). However, 65.2% of HCWs believed that the vaccine was necessary for them. This was slightly higher in physicians (71.8%) than in nurses (61.7%). Thirty-seven percent (37%) of HCWs believe that the COVID-19 vaccine has fatal side effects. This was more obvious among nurses (43.7%) than physicians (24.3%) (*p* < 0.001). The majority of HCWs (77.6%) stated that they needed more information about the vaccine, especially (86.5% of physicians and 72.9% of nurses) (*p*=0.001). The propaganda of official media was trusted by 32.8% of HCWs, where nurses showed a significantly higher percentage than physicians (36.9% and 25%, respectively) (*p*=0.010). Concerning the vaccine's clinical trials, only 38.6% of HCWs believed that the vaccine was fully evaluated by clinical trials (46.8% of physicians and 34.2% of nurses) (*p*=0.009). 50.8% of HCWs stated that they will get the vaccine in the future (67.9% of physicians and 41.7% of nurses) (*p* < 0.001). Besides, 47.5% of them will advise their family members to get the vaccine (56.4% of physicians and 42.7% of nurses) (*p*=0.006). On the other hand, 59.6% of physicians will take their children to get COVID-19 vaccination versus 35.3% of nurses (*p* < 0.001).

As presented in Table 8, medical specialists were the most commonly reported trustworthy source of information about the COVID-19 vaccine.

Healthcare workers' attitudes post-pandemic are presented in Table 9. Periodical administration of the COVID-19 vaccine was reported by 59.4% of HCWs; there was no significant difference between nurses and physicians. Similar behavior was detected toward influenza vaccine (68.1% of HCWs). Nurses reported significantly more preference to take pneumococcal vaccine annually than physicians (47.1% and 26.3%, respectively) (*p* < 0.001). Reducing the frequency of going to crowded places was reported more among physicians (89.7%) than nurses (79%) (*p* = 0.004). In addition, desire to keep washing hands frequently was significantly higher among physicians (93.6%) than nurses (85.1%) (=0.008). Other postpandemic attitudes reported by HCWs included keeping up with exercises frequently (72.7%), trying to lose weight (67.4%), and keeping wearing masks outdoors (66.7%). There was no significant difference between physicians and nurses regarding these attitudes.

As presented in Table 10, the mean age of HCWs accepting to take the COVID-19 vaccine was significantly higher than those refusing (32.6 ± 7.9 and 30.8 ± 7.2, respectively) (*p*=0.011). Males showed a significantly higher acceptance for the vaccine than females (53.4% and 31.7%, respectively) (*p* < 0.001). Regarding the occupational group, 55.1% of physicians accepted to take the vaccine versus 33.2% of nurses (*p* < 0.001). Concerning the educational level, the highest acceptance of the vaccine was recorded among HCWs having medical doctorate (57.1%) and master's degree (56.2%) (*p*=0.001). Besides, HCWs accepting the vaccine had significantly larger mean duration of work experience (8.8 ± 6.8) than those refusing the vaccine (7.4 ± 6.3) (*p*=0.002).

As shown in Table 11, there was a statistically significant relation between vaccine acceptance and medical status of the HCWs.

There was no significant relation between vaccine acceptance and HCWs' work experience with COVID-19 as shown in Table 12.

Logistic regression analysis was conducted for factors affecting vaccine acceptance. A backward conditional method was used. Factors entered into the model were age, gender, occupation, the level of education, and duration of work experience. Gender and occupation were found to be predictors of COVID-19 vaccine acceptance as presented in Table 13.

## 4. Discussion

The current study investigated whether healthcare workers would accept COVID-19 vaccines since they are on the front lines of pandemic response and are more susceptible to infection.

The main findings showed that 40.8% agreed to receive the vaccine and 59.2% disagreed. The acceptance percentage among physicians (55.1%) was much greater than that of nurses (33.2%). Similar to another study, in which 46% of the participants were either totally agreeing or somewhat agreeing to take the vaccine, despite the fact that a higher percentage of participants intended to accept the vaccine, the overall acceptability of the responses is considered low [34]. The lack of safety, fear of genetic mutation, and new technology, as well as the belief that the vaccines are ineffective, were the main reasons for disagreement. The existence of comorbidities or chronic conditions, as well as the age of healthcare personnel (older participants tend to approve more), were the key factors influencing COVID-19 acceptability.

In terms of vaccination acceptance, a survey of 613 Congolese healthcare workers (HCWs) found that only 28% of participants would accept COVID-19 vaccination [41]. A study from France included 3259 people who completed an online questionnaire, and it found that nearly 3/4 of them (77.6%, 95 percent confidence interval 76.2–79 percent) would take the vaccine. Healthcare professionals were 81.5% likely to get vaccinated, compared to 73.7 percent of non-healthcare workers [42].

Furthermore, research performed in the United States found that only 30% of participants would prefer not to obtain the vaccination as soon as it becomes available [41, 43].

The intention to accept COVID-19 vaccination among Egyptian HCWs is low relative to studies from western countries but higher than African research. This could be explained by participants' misconceptions obtained from social media as a source of knowledge [34]. Besides, the variety of professions represented among the respondents in this study may have influenced our findings, as a large portion of respondents (39.8%) were medical students, who had a lower degree of expertise than doctors. In addition, since most of the other compared investigations were completed in early 2020, the time effect can be added to the discrepancy between the results of the current study and the other compared research. It was the peak of the pandemic, and many people believed that the vaccine was the magical cure to infection control, which influenced the responses of those who took part in many surveys.

According to the results of this survey, a higher percentage of the participants intended to reject the vaccine, indicating low acceptability among participants. Vaccine apprehension among Egyptian HCWs could be a key stumbling block in the country's vaccine acceptance decision. Along with a global survey of 13,426 participants conducted in 19 countries to investigate the possible acceptability of the COVID-19 vaccination, 71.5% would like to take the vaccine in some way. Despite the fact that they reported a higher percentage of vaccine acceptability, variances in acceptance ranged from 80% in Asian countries to less than 55% in Russia [44]. As a result, it is unsurprising that vaccination acceptability is low in Egypt, given the global prevalence of vaccination hesitancy.

In terms of the vaccination type, it was discovered that 21.9% of participants preferred mRNA-based vaccines (Pfizer/BioNtech) and 21.2% preferred Oxford/Astra Zeneca if available, while the remaining participants were divided among the other vaccine types and refused vaccination. It is uncertain why Pfizer vaccines are more likely to be accepted by participants than other vaccines. It could be related to the participants' trust in the brand and the transparency with which information about their vaccine is presented in public.

The current results demonstrated that medical specialists and medical literature were the most commonly trusted sources of knowledge for the participants; 70.1% and 38.6%, respectively. Fortunately, these sources are preferable to be the source of knowledge owing to the disinformation that can be spread to the public (conspiracy theory) by some social media posts claiming that the use of an mRNA-based COVID-19 vaccination can alter the DNA of the population [34, 45]. This could contribute to vaccination acceptance hesitancy.

To investigate the factors that can affect the acceptance of the vaccine in the present study, regarding the history of chronic diseases, 20% of HCWs suffered from chronic illnesses (16.7% of physicians and 21.7% of nurses). The most frequently reported medical conditions were diabetes and hypertension, which were significantly reported by nurses than physicians. This was similar to another study that demonstrated a significant correlation regarding age and the history of chronic diseases using univariate regression analysis [34]. This may be explained by the fear of those groups about the impact of COVID-19 on their comorbidities as reported in many studies indicating that diabetes mellitus [46], chronic hepatic and renal diseases [47], and multiple comorbidities, especially neurologic ones, can increase both morbidity and mortality in COVID-19 patients [48].

Regarding vaccine safety, only 32.8% of HCWs believed that it was safe. In another study, concerns regarding the vaccine's safety were expressed by 57 percent of study participants [34]. Similar findings were observed in many places, and a number of variables linked to vaccine reluctance around the world included plenty of questions regarding the vaccine's safety and efficacy [49], which persisted even after the SARS-CoV-1 pandemic [50]. Fear of such a rapid public release of the vaccine due to a lack of proper research and a lack of research on the Arab population has led to increased doubt about the benefits of receiving newly formed vaccines.

Our study shows that gender and occupation were identified as predictors of acceptance of the COVID-19 vaccine. Comparable to another study claiming that age, education, and ethnicity were the main determinants of COVID-19 vaccination acceptance and that models based on sociodemographic characteristics could accurately estimate COVID-19 vaccine acceptance [51], Additionally, there are conflicting studies on the impact of gender, with some claiming that males were more likely to accept the vaccine than females [52], while others claim that female acceptance was higher [53].

## 5. Conclusion

In Egypt, vaccination apprehension is frequent among HCWs, and this could be a serious barrier to vaccine acceptance among the common society. Campaigns to raise awareness on the importance of vaccines are urgently needed.

## 6. Recommendation

There is a need to clarify the incorrect conceptions and misconceptions that have arisen as a result of the use of social media. Clear communication between national government officials and HCWs is helping to build confidence. All of this can be accomplished by explaining how the vaccine works, its level of effectiveness, its safety, and predicted side effects, as well as the vaccine uptake mechanism (doses and site). HCWs should be given lectures prepared by trusted medical leaders who can address any questions they may have.

### 6.1. Limitation of the Study

A cross-sectional, descriptive study design was used in this study with the advantages that the study has several outcomes, has control over measurements with a short duration, could yield prevalence, and is relatively quick and inexpensive.

From its disadvantages are that it gave a background on the perception and vaccine acceptance of COVID-19 vaccination among healthcare workers in a certain period during this study.

## Figures and Tables

**Table 1:** Demographic data of study participants.

Demographic and general information	Freq.	%
Age (in years) (mean ± SD)		
Gender		
Male	189	41.9
Female	262	58.1
Marital status		
Single	158	35.0
Married	267	59.2
Divorced	18	4.0
Widow	8	1.8
Residence		
Rural	94	20.8
Urban	357	79.2
Occupation		
Physicians	156	34.6
Nurses	295	65.4
The number of household members (excluding participant) (median, range)	4	1–10
Department		
Surgical departments	64, 33	21.5
Medical (nonsurgical) departments	188	41.7
ICU departments	82	18.2
Isolation department	44	9.8
Emergency department	47	10.4
Education level		
Nursing Institute	82	18.2
Bachelor	136	30.2
Diploma	123	27.3
MSc	89	19.7
Medical doctorate	21	4.7
Work experience in years (mean ± SD)	8 ± 6.5	1–42

**Table 2:** Medical history of studied healthcare workers

	Total (451)	Physicians (156)	Nurses (295)	*p* value
Suffered from any chronic illness	90 (20.0%)	26 (16.7%)	64 (21.7%)	0.204^¥^
Type of illness				
Diabetes mellitus	40 (8.9%)	13 (8.3%)	27 (9.2%)	0.770^¥^
Hypertension	46 (10.2%)	12 (7.7%)	34 (11.5%)	**<0.001** ^¥^ ^*∗*^
Chronic liver diseases	3 (0.7%)	0 (0.0%)	3 (1.0%)	0.554^§^
Chronic kidney diseases	1 (0.2%)	1 (0.6%)	0 (0.0%)	0.346^§^
Bronchial asthma	13 (2.9%)	5 (3.2%)	8 (2.7%)	0.772^§^

^*∗*^Statistically significant at *p* value <0.05. ^¥^Chi-square test. ^§^Fisher's exact test.

**Table 3:** HCWs' work experience with COVID-19.

	Total (451)	Physicians (156)	Nurses (295)	*p* value
Participated in the prevention and control of the pandemic	272 (60.3%)	109 (69.9%)	163 (55.3%)	**0.003** ^*∗*^^¥^
Received other vaccines in the past 3 years	218 (48.3%)	84 (53.8%)	134 (45.4%)	0.089^¥^
Received seasonal influenza vaccine in the last 3 years	201 (44.6%)	63 (40.4%)	138 (46.8%)	0.194^¥^
Got COVID-19 infection	239 (53.0%)	86 (55.1%)	153 (51.9%)	0.509^¥^
Method of diagnosis				
Clinical presentation	163 (36.1%)	76 (48.7%)	87 (29.5%)	**<0.001** ^*∗*^^¥^
Laboratory investigation	91 (20.2%)	35 (22.4%)	56 (19.0%)	0.385^¥^
Chest CT	109 (24.2%)	43 (27.6%)	66 (22.4%)	0.221^¥^
PCR	68 (15.1%)	32 (20.5%)	36 (12.2%)	**0.020** ^*∗*^^¥^

^*∗*^Statistically significant at *p* value <0.05; ^¥^chi-square test.

**Table 4:** Assessment of knowledge, attitude, and practice towards COVID-19.

		Total (451)		Physicians (156)		Nurses (295)		*p* value
		Yes	No/don't know	Yes	No/don't know	Yes	No/don't know	
16	All age groups are susceptible to COVID-19	417 (92.5%)	34 (7.5%)	150 (96.2%)	6 (3.8%)	267 (90.5%)	28 (9.5%)	**0.031** ^*∗*^^¥^
17	Increase risk of death in elderly patients or patients with chronic illnesses	421 (93.3%)	30 (6.7%)	156 (100.0%)	0 (0.0%)	265 (89.8%)	30 (10.2%)	**<0.001** ^*∗*^^¥^
18	There is a slight mutation in the COVID-19 virus	264 (58.5%)	187 (41.5%)	122 (78.2%)	34 (21.8%)	142 (48.1%)	153 (51.9%)	**<0.001** ^*∗*^^¥^
19	Immunity after COVID-19 infection is for short time	256 (56.8%)	195 (43.2%)	114 (73.1%)	42 (26.9%)	142 (48.1%)	153 (51.9%)	**<0.001** ^*∗*^^¥^
20	Do you believe COVID-19 could be diminishing in summer?	253 (56.1%)	198 (43.9%)	74 (47.4%)	82 (52.6%)	179 (60.7%)	116 (39.3%)	**0.007** ^*∗*^^¥^
21	Do you think COVID-19 will be seasonal pandemic like influenza?	323 (71.6%)	128 (28.4%)	115 (73.7%)	41 (26.3%)	208 (70.5%)	87 (29.5%)	0.472^¥^
22	Do you think COVID-19 will continue to spread periodically?	314 (69.6%)	137 (30.4%)	138 (88.5%)	18 (11.5%)	176 (59.7%)	119 (40.3%)	**<0.001** ^*∗*^^¥^
23	Have you directly or indirectly taken care of the COVID-19 patients?	367 (81.4%)	84 (18.6%)	155 (99.4%)	1 (0.6%)	212 (71.9%)	83 (28.1%)	**<0.001** ^*∗*^^¥^
24	People may be infected with COVID-19 without any symptoms	375 (83.1%)	76 (16.9%)	153 (98.1%)	3 (1.9%)	222 (75.3%)	73 (24.7%)	**<0.001** ^*∗*^^¥^
25	Have you, your family member, or someone you know been diagnosed with COVID-19?	329 (72.9%)	122 (27.1%)	137 (87.8%)	19 (12.2%)	192 (65.1%)	103 (34.9%)	**<0.001** ^*∗*^^¥^

^*∗*^Statistically significant at *p* value <0.05 and ^¥^chi-square test.

**Table 5:** Self-perceived risk of COVID-19 among HCWs.

Do you think you are at risk of getting COVID-19 in the next 1 year?	Total (451)	Physicians (156)	Nurses (295)	*p* value
No I am confident I won't get infected	46 (10.2%)	10 (6.4%)	36 (12.2%)	**0.025** ^*∗*^^¥^
Yes, but I think that I will get mild symptoms which will probably not require hospitalization	220 (48.8%)	90 (57.7%)	130 (44.1%)	
Yes, but I think that I will get moderate symptoms which will probably need hospitalization	98 (21.7%)	34 (21.8%)	64 (21.7%)	
Yes I am concerned that I will get severe symptom which will probably require admission to the intensive care unit	33 (7.3%)	9 (5.8%)	24 (8.1%)	
I believe I already have the disease and I am immune to it (not diagnosed by a test)	31 (6.9%)	10 (6.4%)	21 (7.1%)	
No, I already have recovered and won't get reinfected (diagnosed by a test)	23 (5.1%)	3 (1.9%)	20 (6.8%)	

^*∗*^Statistically significant at *p* value <0.05; ^¥^chi-square test.

**Table 6:** Acceptance of COVID-19 vaccine.

	Total (451)	Physicians (156)	Nurses (295)	*p* value
(26) Do you accept taking COVID-19 vaccine?				
(1) Accept COVID-19 vaccine	184 (40.8%)	86 (55.1%)	98 (33.2%)	**<0.001** ^*∗*^^¥^
(2) Reject COVID-19 vaccine	267 (59.2%)	70 (44.9%)	197 (66.8%)	
(27) If you accept vaccination, where would you like to get vaccination?				
(1) Community vaccination clinic	59 (13.1%)	21 (24.4%)	38 (38.8%)	**0.037** ^*∗*^^¥^
(2) Vaccination campaign organized by hospital	125 (27.7%)	65 (75.6%)	60 (61.2%)	
(28) If you had the opportunity to choose the type of vaccine to take from all the available vaccines, which one will you choose?				
(a) Pfizer	97 (21.5%)	60 (38.5%)	37 (12.5%)	<0.001 ^*∗*^
(b) AstraZeneca	69 (15.3%)	31 (19.9%)	38 (12.9%)	0.05^¥^
(c) Sinopharm	3 (0.7%)	2 (1.3%)	1 (0.3%)	0.276^§^
(d) Johnson	1 (0.2%)	0 (0.0%)	1 (0.3%)	1.000^§^

^*∗*^Statistically significant at *p* value <0.05 and ^¥^chi-square test.

**Table 7:** HCWs' beliefs towards COVID-19 vaccines.

	Total (451)		Physicians (156)		Nurses (295)		*p* value
	Yes	No/don't know	Yes	No/don't know	Yes	No/don't know	
Do you prefer to wait to review vaccine safety profile?	369 (81.8%)	82 (18.2%)	131 (84.0%)	25 (16.0%)	238 (80.7%)	57 (19.3%)	0.388^¥^
Do you believe that COVID-19 vaccine is effective?	145 (32.2%)	306 (67.8%)	64 (41.0%)	92 (59.0%)	81 (27.5%)	214 (72.5%)	**0.003** ^*∗*^^¥^
Do you believe that COVID-19 vaccine is safe?	148 (32.8%)	303 (67.2%)	65 (41.7%)	91 (58.3%)	83 (28.1%)	212 (71.9%)	**0.004** ^*∗*^^¥^
Do you believe that COVID-19 vaccine is necessary for healthcare workers?	294 (65.2%)	157 (34.8%)	112 (71.8%)	44 (28.2%)	182 (61.7%)	113 (38.3%)	**0.032** ^*∗*^^¥^
Do you think that the vaccine will be effective for period of time (at least 6 months)	217 (48.1%)	234 (51.9%)	77 (49.4%)	79 (50.6%)	140 (47.5%)	155 (52.5%)	0.701^¥^
Do you think more time is needed before vaccination should be introduced into use?	291 (64.5%)	160 (35.5%)	97 (62.2%)	59 (37.8%)	194 (65.8%)	101 (34.2%)	0.449^¥^
Do you think COVID-19 vaccine has fatal side effects?	167 (37.0%)	284 (63.0%)	38 (24.2%)	118 (75.6%)	129 (43.7%)	166 (56.3%)	**<0.001** ^*∗*^^¥^
Do you think COVID-19 vaccine will be free of charge?	164 (36.4%)	287 (63.6%)	61 (39.1%)	95 (60.9%)	103 (34.9%)	192 (65.1%)	0.379
Do you need more information about COVID-19 vaccination?	350 (77.6%)	101 (22.4%)	135 (86.5%)	21 (13.5%)	215 (72.9%)	80 (27.1%)	**0.001** ^*∗*^^¥^
Do you trust the propaganda of official media?	148 (32.8%)	303 (67.2%)	39 (25.0%)	117 (75.0%)	109 (36.9%)	186 (63.1%)	**0.010** ^*∗*^^¥^
Do you trust your institution advice?	239 (53.0%)	212 (47.0%)	79 (50.6%)	77 (49.4%)	160 (54.2%)	135 (45.8%)	0.467^¥^
Do you believe that COVID-19 vaccine approved for license has been fully evaluated in clinical trials?	174 (38.6%)	277 (61.4%)	73 (46.8%)	83 (53.2%)	101 (34.2%)	194 (65.8%)	**0.009** ^*∗*^^¥^
Will you get COVID-19 vaccination in the future?	229 (50.8%)	222 (49.2%)	106 (67.9%)	50 (32.1%)	123 (41.7%)	172 (58.3%)	**<0.001** ^*∗*^^¥^
Will you advice your family members to get COVID-19 vaccination?	214 (47.5%)	237 (52.5%)	88 (56.4%)	68 (43.6%)	126 (42.7%)	169 (57.3%)	**0.006** ^*∗*^^¥^
Will you take your children to get COVID-19 vaccine?	197 (43.7%)	254 (56.3%)	93 (59.6%)	63 (40.4%)	104 (35.3%)	191 (64.7%)	**<0.001** ^*∗*^^¥^

^*∗*^Statistically significant at *p* value <0.05 and ^¥^chi-square test.

**Table 8:** Trusted sources of information about COVID-19 vaccine.

Who do you trust offering COVID-19 vaccine information	Total (451)	Physicians (156)	Nurses (295)	*p* value
Official media	64 (14.2%)	13 (8.3%)	51 (19.7%)	**0.010** ^*∗*^^¥^
Medical specialists	316 (70.1%)	116 (74.4%)	200 (77.2%)	0.148^¥^
Relatives and friends	16 (3.5%)	3 (1.9%)	13 (4.4%)	0.134^¥^
Colleagues	46 (10.2%)	20 (12.8%)	26 (10.0%)	0.181^¥^
Medical literature	174 (38.6%)	70 (44.9%)	104 (40.2%)	**0.046** ^*∗*^^¥^
Online media	53 (5.8%)	12 (7.7%)	41 (15.8%)	0.052^¥^

^*∗*^Statistically significant at *p* value <0.05 and ^¥^chi-square test.

**Table 9:** HCWs' attitudes postpandemic (post-COVID-19).

	Total (451)		Physicians (156)		Nurses (295)		*p* value
	Yes	No/don't know	Yes	No/don't know	Yes	No/don't know	
When pandemic ends, will you receive vaccine against COVID-19 periodically if available?	268 (59.4%)	183 (40.6%)	101 (64.7%)	55 (35.3%)	167 (56.6%)	128 (43.4%)	0.094^¥^
Do you prefer to take influenza vaccine annually?	307 (68.1%)	144 (31.9%)	105 (67.3%)	51 (32.7%)	202 (68.5%)	93 (31.5%)	0.800^¥^
Do you prefer to take pneumococcal vaccine annually?	180 (39.9%)	271 (60.1%)	41 (26.3%)	115 (73.3%)	139 (47.1%)	156 (52.9%)	**<0.001** ^*∗*^^¥^
Will you try to reduce the frequency of going to crowded places?	373 (82.7%)	78 (17.3%)	140 (89.7%)	16 (10.3%)	233 (79.0%)	62 (21.0%)	**0.004** ^*∗*^^¥^
Will you keep washing hands frequently?	397 (88.0%)	54 (12.0%)	146 (93.6%)	10 (6.4%)	251 (85.1%)	44 (14.9%)	**0.008** ^*∗*^^¥^
Will you keep doing exercises frequently?	328 (72.7%)	123 (27.3%)	111 (71.2%)	45 (28.8%)	217 (73.6%)	78 (26.4%)	0.585^¥^
Will you try to lose weight?	304 (67.4%)	147 (32.6%)	106 (67.9%)	50 (32.1%)	198 (67.1%)	97 (32.9%)	0.858^¥^
Will you keep wearing masks outdoors?	301 (66.7%)	150 (33.3%)	105 (67.3%)	51 (32.7%)	196 (66.7%)	99 (33.6%)	0.853^¥^

^*∗*^Statistically significant at *p* value <0.05. ^¥^Chi-square test.

**Table 10:** Relation between acceptance to take COVID-19 vaccine and demographic data of the studied HCWs.

	Acceptance to take COVID-19 vaccine		*p* value
	Accept (184)	Refuse (267)	
Demographic and general information			
Age (in years) (mean ± SD) (median, range) S.E	32.6 ± 7.9 31.5 22–68 7.9	30.8 ± 7.2 30.0 19–56 7.2	0.011 ^*∗*^^¶^
Gender			
Male	101 (53.4%)	88 (46.6%)	<0.001^*∗*^^¥^
Female	83 (31.7%)	179 (68.3%)	
Marital status			
Single	56 (35.4%)	102 (64.6%)	0.374^§^
Married	117 (43.8%)	150 (56.2%)	
Divorced	8 (44.4%)	10 (55.6%)	
Widow	3 (37.5%)	5 (62.5%)	
Residence			
Rural	34 (36.2%)	60 (63.8%)	0.305^¥^
Urban	150 (42.0%)	207 (58.0%)	
Occupation			
Physicians	86 (55.1%)	70 (44.9%)	<0.001^*∗*^^¥^
Nurses	98 (33.2%)	197 (66.8%)	
Department			
Surgical departments	30 (16.3%)	92 (34.5%)	<0.001^*∗*^^¥^
Medical (nonsurgical) departments	83 (45.1%)	96 (36%)	0.05^¥^
ICU departments	0 (0.0%)	63 (23.6%)	<0.001^*∗*^^¥^
Isolation department	0 (0.0%)	44 (16.5%)	<0.001^*∗*^^¥^
Emergency department	0 (0.0%)	26 (9.7%)	<0.001^*∗*^^¥^
The number of household members (excluding participant) (mean ± SD), (median, range) S.E	3.5 ± 1.0 4, 1–5 0.1	3.7 ± 1.2 4, 1–10 0.1	0.070^¶^
Education level			
Nursing institute	24 (29.3%)	58 (70.7%)	
Bachelor	56 (41.2%)	80 (58.8%)	0.001^*∗*^^¥^
Diploma	42 (34.1%)	81 (65.9%)	
MSc	50 (56.2%)	39 (43.8%)	
Medical doctorate	12 (57.1%)	9 (42.9%)	
Work experience in years (mean ± SD), (median, range) S.E	8.8 ± 6.8 7, 1–42 0.5	7.4 ± 6.3 5, 1–30 0.4	0.002^*∗*^^¶^

^*∗*^Statistically significant at *p* value <0.05. ^¶^Mann–Whitney test, ^¥^chi-square test, and ^§^Fisher's exact test.

**Table 11:** Relation between acceptance to take COVID-19 vaccine and medical history of the studied HCWs.

	Acceptance to take COVID-19 vaccine		*p* value
	Accept (184)	Refuse (267)	
Suffered from any chronic illness	36 (40.0%)	54 (60.0%)	0.863^¥^
Type of illness			
Diabetes mellitus (40)	20 (50.0%)	20 (50.0%)	0.215^¥^
Hypertension (46)	14 (30.4%)	32 (69.6%)	0.131^¥^
Chronic liver diseases (3)	0 (0.0%)	3 (100.0%)	0.273^§^
Chronic kidney diseases (1)	0 (0.0%)	1 (100.0%)	1.000^§^
Bronchial asthma (13)	8 (61.5%)	5 (38.5%)	0.123^¥^

^¥^Chi-square test and ^§^Fisher's exact test.

**Table 12:** Relation between vaccine acceptance and HCWs' work experience with COVID-19.

	Acceptance to take COVID-19 vaccine		*p* value
	Accept (184)	Refuse (267)	
Participated in the prevention and control of the pandemic (272)	118 (43.4%)	154 (56.6%)	0.169^¥^
Received other vaccines in the past 3 years (218)	93 (42.7%)	125 (57.3%)	0.436^¥^
Received seasonal influenza vaccine in the last 3 years (201)	92 (45.8%)	109 (54.2%)	0.054^¥^
Got COVID-19 infection (239)	104 (43.5%)	135 (56.5%)	0.213^¥^

^¥^Chi-square test.

**Table 13:** Logistic regression analysis of predictors of COVID-19 acceptance.

	*B*	S.E.	*p* value	OR	95% C.I. for OR	
					Lower	Upper
Gender (male)	0.717	0.207	0.001 ^*∗*^	2.048	1.366	3.072
Occupation (doctors)	0.696	0.213	0.001^*∗*^	2.006	1.321	3.045
Constant	−1.890	0.419	<0.001^*∗*^	0.151		

^*∗*^Statistically significant at *p* value <0.05.

## Data Availability

Data are available from the corresponding author upon request.
